# Experiences of children with bronchiectasis and their parents in a novel play-based therapeutic exercise programme: a qualitative analysis

**DOI:** 10.1136/bmjopen-2023-078994

**Published:** 2024-07-31

**Authors:** Taryn Jones, Emmah Baque, Kerry-Ann O'Grady, Brooke E Kohler, Vikas Goyal, Gabrielle B McCallum, Anne Chang, Stewart Trost

**Affiliations:** 1 Queensland University of Technology, Brisbane, Queensland, Australia; 2 Griffith University, Nathan, Queensland, Australia; 3 Department of Respiratory, Sleep Medicine Queensland Children’s Hospital, Brisbane, Queensland, Australia; 4 Menzies School of Health Research, Charles Darwin University, Darwin, UK; 5 The University of Queensland - Saint Lucia Campus, Saint Lucia, Queensland, Australia

**Keywords:** Chronic Disease, PAEDIATRICS, QUALITATIVE RESEARCH, Paediatric thoracic medicine, REHABILITATION MEDICINE

## Abstract

**Abstract:**

**Objectives:**

To explore the experiences and perceptions of children with bronchiectasis and their parents regarding an 8-week play-based therapeutic exercise programme.

**Design:**

Qualitative study with inductive content analysis.

**Setting:**

Individual semistructured interviews were conducted. Interview recordings were transcribed verbatim, and coding was guided by the content. Content categories were established via consensus moderation.

**Participants:**

10 parents and 10 children with bronchiectasis aged 5–12 years.

**Results:**

From the perspective of children, the most important components of the programme were fun with friends and being active at home as a family. Parents valued the community-based sessions, perceived the programme to be engaging and motivating. Parents perceived improvements in their child’s endurance, coordination and physical activity level. They described the home programme as fun but noted that finding time was difficult. Both parents and children thought that in-person exercise sessions would be better than exercise sessions delivered online.

**Conclusions:**

Children who participated in the play-based exercise programme, found it fun, motivating and accessible. Parents perceived positive impacts on fitness, coordination and physical activity.

**Trial registration number:**

The trial was registered with, Australian and New Zealand Clinical Trials Register (ACTRN12619001008112).

**STRENGTHS AND LIMITATIONS OF THIS STUDY**

- This study included children as participants who expressed unique opinions about their participation in the physical activity programme highlighting the importance of their inclusion in research focusing on their lived experience.
- Collaborating with families and codesigning research projects is a current research priority area for children and young people with bronchiectasis.
- This study had a relatively small number of participants, but saturation of data was achieved from the 10 parent–child dyads.

## Introduction

 Bronchiectasis unrelated to cystic fibrosis is a chronic lung disease that impacts the daily lives of children, including their schooling, play and overall well-being.^1^,^4^ This pulmonary disorder is diagnosed by identifying the presence of abnormal bronchial dilatation using high-resolution chest CT in the presence of clinical symptoms.^5^,^7^ Children present clinically with a persistent wet cough with or without shortness of breath and poor exercise tolerance.^3 6 8^ The pathology can alter mucociliary clearance creating a cycle of inflammation and infection which can lead to pulmonary exacerbations.^9^,^11^ The frequency of exacerbation is the only known predictor of long-term decline in lung function in children with bronchiectasis.^10^ As the global prevalence of bronchiectasis rises, it is recognised as an important cause of chronic respiratory disease, morbidity and healthcare utilisation.^12^,^15^

The management of bronchiectasis uses a multidisciplinary approach. In children, its goals include improving quality of life, exercise tolerance and lung function while reducing the number of exacerbations and hospitalisations.^16^,^18^ Guidelines for the treatment and management of bronchiectasis call for regular exercise, not only as a means of improving aerobic fitness and health-related quality life but as a self-management tool to reduce the frequency and severity of exacerbations.^17^ Yet, the available evidence indicates most children with bronchiectasis are insufficiently active for health benefits with only 6% achieving the recommended 60 min of daily moderate to vigorous physical activity (MVPA).^4^

Reasons for physical inactivity among children with bronchiectasis are not well understood. However, developmental delays in fundamental movement skill (FMS) proficiency may be a key contributing factor. In a recent study, only 17% of children with bronchiectasis achieved their age equivalency for locomotor skills while fewer than 9% achieved their age equivalency for object control skills.^19^ Importantly, children achieving their age equivalency for locomotor or object control skills exhibited 41% higher levels of MVPA than children not achieving their age equivalency. Collectively, these findings suggest that children with bronchiectasis would substantially benefit from effective therapeutic programmes that improve FMS proficiency, promote regular physical activity and increase cardiorespiratory fitness. Yet, to date, there is paucity of data on how to achieve this.

The Bronchiectasis: Exercise as Therapy Trial (BREATH) is a multicentre randomised controlled trial (RCT) designed to evaluate the effects of a novel 8-week, play-based therapeutic exercise programme on the frequency of acute exacerbations in children aged 5–12 years with radiologically confirmed bronchiectasis. Secondary aims are to assess the programme’s impact on FMS proficiency, device-measured MVPA, cardiorespiratory fitness, perceived movement competence, health-related quality of life and lung function (forced expiratory volume in 1 s).^20^ Informed by the evidence identifying FMS proficiency as a key determinant of habitual physical activity,^21 22^ the programme focuses on developing and enhancing children’s movement competence, motivation and aerobic fitness through developmentally appropriate, play-based activities or games tailored to the child’s fitness and skill level. The programme comprises a combination of supervised and unsupervised exercise therapy sessions. The supervised component consists of eight 60 min group sessions, completed on a weekly basis, led by a clinical exercise physiologist or physiotherapist. The unsupervised component consists of a home-based, parent-led exercise programme, completed two times per week (~20 min per session), during which children and family members complete two games from their most recent 60 min supervised group session.

While the trial is focused on the primary and secondary outcomes above, it is important for the ongoing development and sustainability of the programme to obtain feedback from participants and their parents/carers. Exploring parent’s and children’s perspectives on the programme provides valuable insight into the utility of the programme and drives action required for scale-up and implementation in clinical and community settings. Therefore, the objective of this study was to explore the experiences and perspectives of children with bronchiectasis, and their parents/carers, after participating in the BREATH play-based therapeutic exercise programme.

## Methods

### Participants

Participants for this study were children enrolled in the BREATH RCT and their parents/carers. To be eligible, children must have been randomised to the exercise programme and participated in at least one exercise session.

### Interview guides

Separate interview guides were developed for children and parents (see online supplemental files 1 and 2). The interview guides included questions related to the acceptability of the programme, how it could be improved and related perceptions of the supervised group exercise sessions and the supplemental unsupervised home-based exercise sessions.

### Data collection

Participants completed a single interview via videoconference with a researcher (BEK) not involved in the delivery of the exercise programme. The child interviews were conducted with a parent present or nearby. Interviews continued until no new insights were identified and key concepts became repetitive.^23^ Interviews were digitally recorded, transcribed verbatim, checked for accuracy against the original recording and saved for subsequent analysis. The transcriptions were deidentified and assigned a unique study identification number.

### Data analysis

Data from the interviews were analysed using content analysis with an inductive approach.^24 25^ Transcripts were read and re-read by a member of the research team (TJ) to guide the establishment of a codebook (see online supplemental file 3). Common phrases, words and content from the transcripts formed an initial draft of the codebook which was subsequently reviewed and updated by the research team (TJ, EB, K-AO’G and ST). To test the reliability of the coding scheme, two-parent and two child transcripts were randomly selected and independently coded by two researchers (TJ and EB). Once the codebook was finalised, a member of the research team (TJ) coded the remaining child and parent transcripts. After all transcripts were coded the initial code groupings were discussed by members of the research team (TJ, EB and ST) and collated to form subcategories and final content categories.^26^ Data were managed with NVivo V.12 (QSR International).

### Patient and public involvement

The parents of children involved in this study initiated discussions with their respiratory physicians regarding participation in the intervention component of the RCT. These physicians, who are part of the research team, recommended a postintervention qualitative study to investigate the experiences and perceptions of the participating families. Two parents who participated in the intervention sessions were asked about the study’s value and the potential effectiveness of conducting interviews via videoconference. Participants were not involved in recruitment or dissemination plans.

## Results

### Participant characteristics

From the 17 families eligible to participate, 10 parent–child dyads provided consent and completed interviews. Six families could not be contacted, and one family declined due to a busy schedule. Children were aged from 5 to 12 years (median age=8.2 years, IQR IQR=5.7–9.8). Four of the 10 children were females. All children interviewed had completed seven or eight supervised group exercise sessions. Parent interviews ranged from 21 to 46 min in duration (mean 31±7.2 min) and child interviews ranged from 11 to 19 min in duration (mean 15.5±2.5 min). The annual household income for families was well distributed across low to high income and ranged from US$26 000 to over US$200 000. Parental education ranged from not finishing high school to completing postgraduate qualifications.

#### Content categories: children

Children provided perspectives on the supervised group sessions, unsupervised home-based programme and recommendations for future programmes. The final content categories were having fun with family and friends; being active at home as a family, and; a preference for in-person sessions. Illustrative quotes from participants are presented below for each of the content categories.

#### Fun with friends and family

Children described the face-to-face group sessions and the games as fun. Children frequently talked about specific games such as balloon tennis or hopscotch they perceived to be fun. Most children indicated that they would like to repeat the BREATH programme again.

I thought they were really fun, and I liked how they were different ones each week and sometimes some were the same… I liked doing the hopscotch game. We went outside and did this ring toss, and the rings were really heavy. I liked that too. Ch03

They were fun, and they involved running around a lot and throwing and kicking and stuff. Ch06

They were pretty fun… the one where I do the ball. That was really fun. Ch02

Children valued having other children participate in the exercise sessions. They especially liked when their siblings or friends participated.

…you can be with people that you know… (therapist) was really nice. Ch01

I wasn’t alone… I could compete with my brothers. Ch06

Why was it fun? ‘Because he (brother) got to do activities too and he does that balloon one too… Ch07

It was a bit better because I wasn't just doing all the activities all myself. Ch03

#### Being active at home as a family

Children’s responses regarding the home programme were brief in comparison to their conversations about on the supervised group programme. Children primarily spoke about their siblings and parents’ involvement and described the games included in the home programme as fun.

You can play with your siblings if you’re at home… Sometimes my brother joined in. It was fun. Ch01

There was balloon tennis. For balloon tennis, mum and (sister). For the yoga poses, dad and mum. I liked having my family involved. Ch09

Well, sometimes (brother) would do it with me and mum would sometimes do a little bit and watch… Yeah, I liked it. It did get tiring for some stuff like doing - like in the hallway, going up and down doing like frog jumps. Ch03

#### In person is better than online

Children offered suggestions for future programmes regarding the mode of delivery, use of technology and recommendations for future programmes. Most (but not all) children expressed a preference for the supervised exercise programme component to be delivered face to face rather than ‘online’ or through an exercise ‘app’. However, for the home programme, children thought technology could be useful.

…online, for the for the actual game sessions, no. Ch02

It would be kind of like strange because you couldn’t really—you wouldn't really be able to demonstrate too well and it’s kind of glitchy. Ch03

Yeah, an app would be cool and useful. It would probably have like - like you could like hold it in your hand and it would count how many steps you've done and could somehow sense your heart rate. Just like a phone or a tablet. Ch03

App with activities, like daily activities, and then it would have like a couple of weekly. Ch02

You’d get to watch the activities then do them. Ch01

### Content categories: parents

Parents provided perspectives on the supervised group sessions, unsupervised home-based programme, perceived impact of the programme on their child and ideas for future programmes. The final emergent themes were an engaging and motivating programme; parents’ perceptions of programme impact family and friends are important; location, location, location; the home programme was fun but finding the time was hard, and; apps are fine for home, but face-to-face sessions are preferred. Illustrative quotes from participants are presented for each content category.

#### An engaging and motivating programme

Parents universally expressed positive feelings about the BREATH programme. Like the children, they thought the exercise sessions were fun and said their child enjoyed the programme. Parents valued the variety of games and activities included the programme and felt that supervised exercise sessions were well structured and organised. They perceived that the rapport with the therapist and the variety of games motivated and engaged their children to participate in the exercise sessions.

It was all very engaging, and she really was motivated by the games because the games were fun… I think that the venue that we were in was so—like something that we didn’t expect and just the fact that she is in this massive hall full of games and equipment. Par04

It motivated him and got him interested in doing different things and that, so I thought it was quite good. All different levels of stuff, like it wasn’t just the same, repetitive things, it was all different stuff… Good variety of activities as well, it would be different each week, it wasn’t repeating in the same sort of thing each week. Par06

She did it very well, because I think she’s loving all those activities, that’s why, yeah… I think all the activities basically, the whole exercise I think, because she loves to play, so that’s why I think she enjoyed those exercises. Par08

#### Perceptions of programme impact

Parents enthusiastically talked about the changes they observed in their child after completing BREATH. Parents reported increased fitness and/or endurance, improved coordination and greater participation in physical activity.

When he plays baseball, he used to get really, really tired playing baseball. He would be so puffed out after doing one innings of baseball and sometimes he’d get that tired he’d have a meltdown because he’s autistic. But now he plays the whole two-and-a-half-hour game without really having a break or having a meltdown. Par01

Especially like when his cousins come over, they will just run through and around the house and up and down the house for hours on end, whereas before he potentially wouldn’t have done that. Par05

It definitely helped her coordination because now she can do hopscotch. She’s better at aiming with her throws now… I think it’s helped her confidence a little bit too actually. Yes, so even when we're just playing games on the weekend and stuff like that her coordination has gotten a lot better. Par07

His coordination has definitely improved, like the hand eye coordination, bouncing balls and hitting things with rackets. A bit of improvement there, that’s for sure… Just in how he plays here at home. Whereas before he'd maybe get over it pretty quickly because he wasn't that great at it, he had a bit more skill. Par02

But to do the weekly exercise program and then see the improvement in him and since then it’s almost like it was a—it was like a trigger for him. So, he now runs better. He plays better. He throws balls. He kicks balls. He’s a lot more physically coordinated that he was and yeah that was probably one of the big takeaways for us and something that we’ve continued to encourage at home. Par05

I think she’s more active now, but I didn’t notice any changes but she’s not getting tired easily basically. So, that also help her, all those exercises. Par08

#### Family and friends are important

Parents valued the participation of siblings and friends in BREATH. Parents said that it helped their child feel more confident in the initial sessions and made the programme more enjoyable overall.

When we were told that we could include the siblings, you know sometimes people say that, but they really meant it. So, like I said, (sibling) still asks to go to the sessions, her little brother. I think it just made it more fun, having her sibling there. Par03

Participating with his siblings, he’s not used to, but it got really good because they become closer. Him and his brother I know are really close because they’re so close in age, but he got to show (sister) how to play. Par01

It was good that there were other kids there. He liked that. I did notice that… he loves social interaction, absolutely thrives off it, and if he can find a friend and someone to play with, and someone that likes his games, he’s very happy. Par02

I think having his sister there made it a fun family experience… (Sister) loved it as well. She just—yeah, she was excited as he was to go there every time. There was another little boy there who had bronchiectasis. Yeah, it made it—it was almost like they saw it as a play date…it was good for them all to do it, I think. Par05

#### Location, location, location

Parents liked that the supervised exercise sessions were delivered in community halls. They valued the spacious venues and the proximity to their home or their child’s school.

The community centre was actually really good because I had never been there before but the fact that it was all inside meant that we didn't have to stop because of the rain. Par07

The location is very convenient for us because it’s only 15 minutes away from us, so it’s very convenient, I cannot complain on that one. We don’t need to travel far, because the option is either go to the other, I think the hospital, right? vs the community centre, I prefer the centre because I know it’s only 15 minutes away from our place. Par08

…that’s perfect location. It was only just up the road from us, it wasn’t a big push to get there. Like I finished work and got to day care to pick him up from after school care and then got there generally early most days. Par09

#### The home programme was fun but finding the time was hard

Parents described the home-based programme as fun. They liked the variety of activities and games included and thought that the frequency and duration were appropriate. They found the instructions helpful and easy to follow. Parents liked that the home programmes could be completed with equipment they had at home.

Still to this day we’ve got a folder where we’ve kept them, and I still have to buy balloons because they’re like balloon tennis is their favourite. They love to play it, like all the time, down my hallway, everywhere. Par01

I like that it gave us some of those activities and things that he did, because some of them he really enjoyed in the moment. So, it was nice to actually have a copy of how to do it and how to set it up and stuff like that. Par02

They will play that game as a matter of course. So yeah—and again it’s—it’s seeing how the strategies or the activities they were doing in class, for want of a better word can be—can just become embedded at home and taking five min to play the balloon game or taking five min to go downstairs and kick a football around or do something. So yeah, it was good. Par05

However, parents described some barriers to completing the home programme. A few parents acknowledged that the home programme was not always a priority. Parents commented that lack of time or their own lack of motivation was a barrier to doing the home exercise component.

We knew what we had to do. It’s more home management of finding the time to do it… there was nothing we disliked. It’s just our innate laziness trying to find times to do the things. Par05

We didn’t do it as often as we should and that’s because of the time… We always did it once before because obviously, the day before, we were going to the next session… we couldn’t do it very often because it’s just too much other things, you know? Par04

I tried begging, I tried pleading. He’s not a fan. As soon as it was called homework, he was very much not interested. Even with the encouragement of the stickers and the whole getting to show off the next time when we went there anything. He was just yeah—he was not very interested in doing it at all. Par02

#### Apps are fine for home, but face-to-face sessions are preferred

Parents provided feedback and suggestions in relation to the mode of delivery and use of technology. There were strong opinions that exercise sessions delivered through a digital platform such as telehealth would not work for their child since parent involvement was crucial. Nevertheless, parents saw value in the use of an online platform or app for the management of the home-based exercise programme.

I think if it was over a Zoom call or anything like that, he would just not be so engaged. So, I kind of liked the fact that it had real people. Par09

Personally, I don't think it would probably work for us…given that that’s just not his thing, doing it like over the phone or telehealth or whatever. Maybe an app would be all right. But it'd still need that face-to-face, I think, interaction, with the actual going to a group and doing that. I think it needs that. Par02

Telehealth would not work ever with (child), no way. We did the dance Zoom classes during the lockdowns and yeah, you know… Oh, she loses the interest like you know, she can just move away herself from the situation. Par04

…maybe if you had an app or something for the older kids where they can just do it on their own maybe, so they didn't have to have mum and dad there or something. Par07

…if we have an app to basically listed all the exercises that we needed to do for a specific day, I think that would be easier instead of the paper base. Especially we’re now on modern technology as well. Par08

## Discussion

Our study explored children’s and parent’s experiences and perceptions of an 8-week developmentally appropriate play-based therapeutic exercise programme for children with non-cystic fibrosis bronchiectasis (BREATH programme). Children and parents provided unique yet complementary perspectives about the BREATH programme. Children thought that including family members and friends in the programme made it more engaging. They valued being physically active at home with family members and preferred in-person exercise sessions to telehealth or online sessions. Parents expressed broader viewpoints than children. Parents described BREATH as an engaging and motivating exercise programme and felt that it had visible positive impacts on their child’s cardiovascular fitness, coordination level and participation in physical activity. Like children, parents indicated a preference for face-to-face sessions over telehealth or app-based exercise programmes. The community-based location and inclusion of family members and friends were considered important strengths. They described the supplemental home programme as fun but acknowledged that finding time to complete the programme was challenging.

The delivery of the programme in readily accessible community-based venues such as council halls was highly valued by parents. Therapeutic exercise programmes are typically delivered in health services, outpatient settings or academic institutions. Thus, parents’ strong endorsement of community halls as a venue for delivering the programme represents an important finding. The families’ preference for exercise programmes delivered locally is consistent with the results of a recent qualitative study that identified supportive physical activity environments as a facilitator of physical activity in children with bronchiectasis.^27^ In this study, parents liked that the community venues were close to home or school, they felt that it was an accepted place where exercise occurs and appreciated the physical space inside the venues. Multiple systematic reviews highlight that physical environmental factors are consistently associated with physical activity.^28^,^31^

Both children and parents thought that the inclusion of siblings and friends in the exercise sessions was fun and motivating, especially at the start of the programme. These findings are consistent with the results of a recent systematic review of 26 qualitative studies exploring children’s perspectives on what they like about physical activity, why it is important and the factors that influence their physical activity.^32^ Although the studies included in the review focused on healthy, typically developing children, being active with friends and being encouraged by their friends as salient influences on children’s physical activity. Being physically active with their families and parental support were also identified as important influences. In a different study, children and young people with cystic fibrosis were a subset of participants interviewed to explore their perceptions of physical activity.^33^ Children with cystic fibrosis reported that they enjoyed physical activity and linked it to health benefits. Similar to this study, they identified peers and family as enablers of physical activity. Collectively, the findings from these studies support the concept that making therapeutic exercise programmes open to family members and friends is an effective strategy to increase enjoyment, engagement and support motivation.

Parents perceived that their child directly benefited from participating in the programme. Parents openly talked about visible improvements in their child’s endurance, level of coordination and physical activity participation. Previous exercise studies in children with bronchiectasis tend to focus on specific activities or components of movement such sit to stand,^34^ balance^35^ and walk testing.^36^ In a different approach to activities and exercise, a recent study investigated the efficacy of aerobic video game exercises and breathing video game exercises in children with bronchiectasis.^37^ The parents’ observations from our study reflect the goals of the BREATH programme which focuses on developing and enhancing children’s confidence and motivation to engage in physical activity through developmentally appropriate, play-based activities targeting aerobic fitness and FMS. The perceived improvements in coordination and endurance are consistent with the results of the BREATH pilot RCT.^22^ In this study, relative to usual care controls, children receiving the play-based therapeutic exercise programme exhibited significant improvements in cardiovascular fitness, locomotor skills and object control skills.^22^ The perceived increase in physical activity after completing the programme is consistent with the findings of a previous study conducted in children with bronchiectasis which reported FMS proficiency to be associated with higher levels of daily MVPA.^4^ While the empirical evaluation of the BREATH programme on frequency of exacerbations, aerobic fitness, FMS, physical activity, quality of life and lung function is ongoing, the findings of our qualitative study indicate that the programme is on track.

When asked to consider a hypothetical scenario where the BREATH exercise programme was delivered via the internet or smartphone, both parents and children indicated a preference for face-to-face exercise sessions over telehealth. Digital healthcare encompasses telehealth, phone contact, text messaging, digital applications (or apps) and is increasingly part of the healthcare landscape.^38^ Unsurprisingly, there was a sharp increase in digital healthcare during the COVID-19 pandemic which has prompted discussion as to its continued role and future innovations.^39^ In the current study, parents and children clearly expressed their preference for face-to-face exercise sessions, citing the positive experience of engagement with other children and the therapist. Parents and children did, however, see a role for of an app or online platform for completing the supplemental home exercise programme, in which many parents described as difficult to prioritise. Families preferred an app or mobile-health (m-health) platform that would be specifically tailored to children with bronchiectasis. It is important to consider these preferences when designing exercise programmes to increase fitness, movement competence and habitual physical activity in children with bronchiectasis.

This study has both strengths and limitations. A strength of the current study is the inclusion of children as participants. Children expressed unique opinions about their participation in the BREATH programme which highlights the importance of their inclusion in research focused on their lived experience. Collaborating with families and codesigning research projects is a current research priority area for children and young people with bronchiectasis.^40^ The study followed established content analysis guidelines and used a rigorous collaborative process for data analysis. Limitations include the relatively small number of participants interviewed and the omission of perspectives from the therapists delivering the programme who could be included in future research. Children who participated in the exercise sessions but did not participate in the interview study may have offered different perspectives. However, saturation of data was reached from the parent–child dyads that were interviewed and the views of the therapists were not a focus of the study.

In summary, we explored the experiences and perceptions of families who participated in an 8-week play-based therapeutic exercise programme to reduce the frequency of acute exacerbations in children with bronchiectasis. The findings suggest that the children who participated in the BREATH programme demonstrated improvements in fitness, coordination and physical activity participation and found the programme fun, motivating and accessible.

## supplementary material

10.1136/bmjopen-2023-078994 online supplemental file 1

## Data Availability

Data are available on reasonable request.
