# Supplementary material for: Experiences of children with bronchiectasis and their parents in a novel play-based therapeutic exercise programme: a qualitative analysis
Source: BMJ Open. 2024 Jul 31;14(7):e078994. doi: 10.1136/bmjopen-2023-078994 (PMC11293381; doi:10.1136/bmjopen-2023-078994)
Supplement: online supplemental file 1 [file bmjopen-14-7-s001.pdf]

Supplemental Table 1: Interview Guide developed for children

Topic	Questions and prompts
BREATH exercise program questions	You did the BREATH program at (*interest location*). You were joined by (*insert siblings, friends*).<br>• Tell me what you thought about the games and activities?<br>o What did you like about the games/activities? Why?<br>o What parts did you think were fun?<br>o What didn't you like about the program? Why?<br>o What parts did you think were boring?<br>o What would you change?<br>• What did you think about how long each session went for?<br>• What did you think about the having the sessions at (*insert location*)?<br>• What was it like having your (friend/sibling there)?<br>• Have you noticed any changes to the way your body feels or moves since doing the exercise program?<br>o What are those changes?<br>• Have you noticed that it is easier or harder to keep up with your friends when you are playing?<br>• Do you get tired when you are playing or running around?<br>o Did this tiredness change after you did the games/exercise program?
Home activity program	• What did you think about the home programs?<br>o What did you like about your home programs?<br>o What was fun?<br>o What didn't you like about the home programs?<br>o What was boring?<br>o What did you think about the types of activities?<br>o What did you think about how long your home activities went for?<br>• What would you change about the home programs?<br>• Who did the home programs with you?<br>o What was that like?<br>o How many times a week did you do the home activities?<br>• Would it be helpful to have an app or other online support?<br>o What would that look like?
Future programs	• Would you do the games and exercise program again?<br>• Would you recommend that other children do the games/ activity sessions?<br>o Can you tell my why/why not?<br>• >10yo: How do you feel an ideal program would be delivered?<br>o Group sessions, one-on-one, home-based, combination, remote, online coaching, apps

	○ Would you recommend that friends/siblings be included<br>○ Timing. Before or after school, on the weekends, or in the holidays?<br>○ Frequency. More/less than once a week<br>○ Types of activities<br>○ Setting. At home, in the community, at a health centre.<br>• <10yo: Would you do the BREATH program again? Would you want to try anything different?<br>• >10yo: Do you have any thoughts how this would work if we delivered this online via a smart phone or iPad?
Sweeping question	Is there anything else you would like to add before we finish?

Supplemental Table 2: Interview Guide developed for parents

Topic	Questions and prompts
BREATH exercise program questions	Your child participated in the bronchiectasis research project that included games/exercise sessions at (*interest location*). They were joined by (*insert siblings, friends*).<br>• Tell me what you thought about the BREATH program?<br>o What did you like about the program?<br>o What could we have done better?<br>• What did you think about the types of activities?<br>• What did you think about the length of each session? *(approx. 1hr)*<br>• What did you think about the length of the whole program? *(8 weeks)*<br>• What did you think about the location?<br>• How did your child feel about participating in the exercise program?<br>o What did they like?<br>o What did they dislike?<br>o What parts of the sessions did you child find easy?<br>o What parts of the sessions did your child find hard or difficult?<br>• Have you noticed any changes in your child's movement skills or coordination level?<br>o What type of changes in movement skills or coordination did you observe?<br>o Did they improve? Did they stay the same? Did they decline?<br>• Have you noticed any changes in their fitness since participating in the games/exercise sessions?<br>o Did their fitness improve? Did it stay the same? Did their fitness decline?<br>• Is there any change to their tiredness or fatigue?<br>o Do they become more fatigued with physical activity? No change? Less fatigued with physical activity?<br>• The BREATH program is designed to include siblings, friends or other children with bronchiectasis. Describe how the inclusion of other children influenced your child?
Home activity program	• What did your home program sessions look like?<br>o Who was usually involved in the home games and activities?<br>o Did you use any particular strategies to manage the home program (e.g. sibling and parent involvement, supervision, competition, rewards, music)?<br>• What did you think about the home programs?<br>o What did you like about the home program?<br>o What did you dislike about the home program?

	o What did you think about the length of each home program?<br>o What did you think about how often you were asked to do the home program?<br>o What did you think about the types of activities?<br>o Was it difficult to motivate you child to do the home program? If so, in what way?<br>o What did you think about the paper handouts you were provided for the home program?<br>• How often did you do the home program?
Future programs	• Describe what you think would be an ideal program.<br>o Group sessions, one-on-one, home-based, combination, remote, online coaching, apps<br>o Timing. Would BREATH be suited closer to the diagnosis of bronchiectasis.<br>o Frequency. More/less than once a week<br>o Length of program, is 8 weeks, too long, too short or the right about of time?<br>o Types of activities<br>o Setting. At home, in the community, at a health centre.<br>o Timing. Before or after school, on the weekends, or in the holidays?<br>• Do you have any thoughts how this would work if we delivered over the internet or smart phone app?<br>• Would a program like BREATH be valuable for other children with bronchiectasis?
Sweeping question	Do you have any other comments you would like to add before we finish?

Supplemental Table 3: Codebook to support content analysis of child and parent interview

Topic	Topic Sub Grouping	Initial Code
BREATH Program	Location	Community
		Home
		Hospital
		Inside
		Other location
		Outside
		Proximity (to home/school)
		Research Centre
		School
		Travel time
	Feelings about program	Adaptable
		Bad
		Boring
		Challenging
		Difficult
		Dislike
		Easier
		Fun / enjoyment
		Games-based
		Good
		Happy
		Hard or harder
		Helpful
		Improve
		Individualised
		Interesting
		Like
		Play- based
		Rapport (therapist, or others at sessions)
		Social / socialise
		Structured
		Variety
	Logistics	Communication therapist
		Communication written
		Equipment support
		Organisation
	Support People	Child
		Friend
		Parent
		Sibling
		Therapist
		Other people (not listed above)
	Timing of diagnosis	Appropriate as was
		Prefer closer to diagnosis
		Prefer further from diagnosis

Changes in participant	Symptoms	Breathless
		Coughing
		Tired / fatigue / exhausted
	Other changes	Ability
		Confidence
		Co-ordination
		Fitness
		Motivation
		No change
		Participation
		Reducing Medicine
		Skills- balance
		Skills- ball
		Skills- exercise (and activities)
		Skills- jumping
		Skills- Play
		Skills- running
		Skills- throwing
		Tried (or trying)
Duration and Frequency BREATH	Frequency F2F Sessions	Appropriate as was
		Prefer more frequent
		Prefer less frequent
	Duration of F2F sessions	Appropriate as was
		Prefer longer
		Prefer shorter
	Duration of BREATH program	Appropriate as was
		Prefer longer
		Prefer shorter
Home program	Frequency of home sessions	Appropriate as was
		Prefer more frequent
		Prefer less frequent
	Duration of home sessions	Appropriate as was
		Prefer longer
		Prefer shorter
	Management of home program	Competition
		Equipment
		Parent involvement
		Reminders
		Rewards
		Sibling involvement
		Supervision
	Sentiment towards home program	Bad
		Boring
		Challenging
		Child autonomy
		Difficult

		Dislike
		Easier
		Fun
		Games-based
		Good
		Happy
		Hard or harder
		Helpful
		Improve
		Interesting
		Like
Future Programs	Mode	Face to face
		Group based
		Individual with therapist
		Using tech (like an APP) for games sessions
		Using tech (like an APP) for home program
	Time /timing	Afternoon
		Day
		Evening
		Holidays
		Morning
		School Term
		Weekend
	Recommend to others	Comment
