# Supplementary material for: Experiences of children with bronchiectasis and their parents in a novel play-based therapeutic exercise programme: a qualitative analysis
Source: BMJ Open. 2024 Jul 31;14(7):e078994. doi: 10.1136/bmjopen-2023-078994 (PMC11293381; doi:10.1136/bmjopen-2023-078994)
Supplement: Supplementary file 2 [file bmjopen-2023-078994.reviewer_comments.pdf]

PEER REVIEW HISTORY

BMJ Open publishes all reviews undertaken for accepted manuscripts. Reviewers are asked to complete a checklist review form (**http://bmjopen.bmj.com/site/about/resources/checklist.pdf**) and are provided with free text boxes to elaborate on their assessment. These free text comments are reproduced below.

ARTICLE DETAILS

**TITLE (PROVISIONAL)**	The experiences of children with bronchiectasis and their parents in a novel play-based therapeutic exercise program: a qualitative analysis.
**AUTHORS**	Jones, Taryn; Baque, Emmah; O'Grady, Kerry-Ann; Kohler, Brooke E; Goyal, Vikas; McCallum, Gabrielle B.; Chang, Anne; Trost, Stewart

VERSION 1 – REVIEW

**REVIEWER**	Dhar, Raja<br>Fortis Hospital Anandapur, Department of Respiratory Medicine
**REVIEW RETURNED**	15-Dec-2023

**GENERAL COMMENTS**	This study explores a novel concept. The methodology of the study and especially the interview guides designed were very nicely done.<br>Hence the study has a lot of potential.<br>However the number of patient family dyads recruited seem insufficient. The drop out of 7 families (out of 17) seems a bit too many. While this is a qualitative study, even then having just a handful of families would prevent you from drawing any inferences which could be generalized to population of children who have bronchiectasis.<br>The interviews while being detailed are probably longer than desired at mean of 31 minutes for parents and 15.5 minutes in children. This would likely explain the number of drop outs.<br>I agree with the authors admission that we should have had the therapists inputs to complete the picture.<br>Hence, I feel this study is not worthy of being published in the BMJ in its current form. However, recruiting more family dyads and making the interviews shorter and crisper esp for the parents is required. Including a short interaction with the therapist would add value. If this is all done I think we will have a study which will be generalizable to a population of children with Bronchiectasis.

**REVIEWER**	Lee, A<br>Monash University , Physiotherapy
**REVIEW RETURNED**	20-Mar-2024

**GENERAL COMMENTS**	This qualitative study is exploring the perspectives of children and their parents of an exercise program (BREATH) for children with bronchiectasis. It complements the quantitative arm of this RCT and provides important perspectives of the utility of the program and factors to consider for the possibility of a broader implementation in the future. It is a well written manuscript, with a clear objective and related well to the existing literature. The

	rationale for the original BREATH study is outlined as is the reasoning for exploring the perspectives of participants. The results reported a clear and comprehensive outline of the findings from both groups of participants in this study, with supporting quotations has been systematically included.<br>Below are some minor suggestions for the authors to clarify.<br><br>Methods: When it states that the transcripts were checked for accuracy, were they returned to the parent or child for this process or checked by the researcher who undertook the interview?<br>Discussion: a systematic review of 26 qualitative studies exploring children's perspectives on what they like about physical activity. Did any of the included studies in this review include children with bronchiectasis or other chronic suppurative lung disease or were they all healthy children?<br><br>Minor point:<br>Discussion: Page 14, line 343, excepted should read accepted instead.

VERSION 1 – AUTHOR RESPONSE

Reviewer 1

Reviewer comment: This study explores a novel concept. The methodology of the study and especially the interview guides designed were very nicely done. Hence the study has a lot of potential.

Author response: We thank the reviewer for their positive comments about the study.

Reviewer comment: However, the number of patient family dyads recruited seem insufficient. The drop out of 7 families (out of 17) seems a bit too many. While this is a qualitative study, even then having just a handful of families would prevent you from drawing any inferences which could be generalized to population of children who have bronchiectasis.

Author response: Thank you for your comments. It is important to note that the seven families did not drop out of our of the study. These families elected not to participate in the qualitative interviews which was a separate voluntary study that occurred after their participation in the intervention arm of the RCT. The goal of qualitative research studies is not to generalise but rather to provide a rich, contextualised understanding of some aspect of human experience through the intensive study of particular cases (Polit & Beck, 2010).
The key principle for assessing the adequacy of the sample size in qualitative research is saturation. Saturation refers to a point at which gathering more data reveals no new theoretical or practical insights. In the current study, our sample of 10 parents and 10 children for a total of 20 interviews was sufficient to reach saturation. This is consistent with the results of recent systematic review addressing the sample size required for qualitative research. The authors concluded that saturation cold be achieved with a range of 9 and 17 interviews (Hennink & Kaiser, 2022). Moreover, the sample size was identified as a study limitation on (Page 11, Line 420 - 422). We also clearly stated that the sample was sufficient for saturation (Page 11, Line 423-424). We have revised the manuscript to make this point clearer: "Interviews continued until no new insights were identified and key concepts became repetitive" (Page 4, Lines 114-115).

Reviewer comment: The interviews while being detailed are probably longer than desired at mean of 31 minutes for parents and 15.5 minutes in children. This would likely explain the number of dropouts.

Author response: Thank you for your concern around the length of the interviews. However, semi-structured interviews typically range from 30 minutes to over an hour. Hence, the duration of the interviews is in keeping with expectations for this methodological approach and similar previously published papers. As noted above, there were no dropouts in the current study and duration of the interviews had no impact on the participant's willingness to participate in the RCT. Further, participants were able to speak for as long as they wished, i.e. there was no time limit, once they felt they had nothing more to add the interviews concluded.

Reviewer comment: I agree with the authors admission that we should have had the therapists' inputs to complete the picture. Including a short interaction with the therapist would add value.

Author response: The purpose of our study was to explore the experiences and perspectives of children with bronchiectasis, and their parents/carers, after participating in the BREATH play-based therapeutic exercise program. As noted on Page 11 Lines 412-413 the views of the therapists delivering the program was outside the scope of the study. We have revised the manuscript: "…and the omission of perspectives from the therapists delivering the program whom could be included in future research." (Page 11, lines 420-421).

Reviewer comment: Hence, I feel this study is not worthy of being published in the BMJ in its current form. However, recruiting more family dyads and making the interviews shorter and crisper esp for the parents is required. If this is all done I think we will have a study which will be generalizable to a population of children with Bronchiectasis

Author response: As stated above, the goal of qualitative research studies is not to generalize to other samples/populations but rather to provide a rich, contextualized understanding of some aspect of human experience through the intensive study of particular cases. Our sample size was sufficient to reach saturation and provided valuable insights into the lived experience of children and families completing our exercise program.

Reviewer 2

Reviewer comment: This qualitative study is exploring the perspectives of children and their parents of an exercise program (BREATH) for children with bronchiectasis. It complements the quantitative arm of this RCT and provides important perspectives of the utility of the program and factors to consider for the possibility of a broader implementation in the future. It is a well written manuscript, with a clear objective and related well to the existing literature. The rationale for the original BREATH study is outlined as is the reasoning for exploring the perspectives of participants. The results reported a clear and comprehensive outline of the findings from both groups of participants in this study, with supporting quotations has been systematically included.

Author response: We thank the reviewer for the positive evaluation of our study and the paper.

Reviewer comment: Methods: When it states that the transcripts were checked for accuracy, were they returned to the parent or child for this process or checked by the researcher who undertook the interview?

Author response: We can confirm that the transcripts were compared against the original recordings by a member of the research team who was not involved in the interview. The following text has been updated: Interviews were digitally recorded, transcribed verbatim, checked for accuracy against the original recording, and saved for subsequent analysis (Page 4, Lines 115-116).

Reviewer comment: Discussion: a systematic review of 26 qualitative studies exploring children's

perspectives on what they like about physical activity. Did any of the included studies in this review include children with bronchiectasis or other chronic suppurative lung disease or were they all healthy children?

Author response: The studies in the systematic review were from a typically developing population. No studies included bronchiectasis, CSLD or other chronic respiratory diseases. The following text has been updated “Although the studies included in the review focused on healthy, typically developing children, being physically active with their families and parental support for physical activity were also identified as important influences” (Page 10, lines 370-372).

Reviewer comment: Minor point: Discussion: Page 14, line 343, excepted should read accepted instead

Author response: Thank you. This has now been amended. (Page 10, line 363)

References:

Hennink, M., & Kaiser, B. N. (2022). Sample sizes for saturation in qualitative research: A systematic review of empirical tests. Social Science and Medicine, 292, 114523. https://doi.org/https://doi.org/10.1016/j.socscimed.2021.114523

Polit, D. F., & Beck, C. T. (2010). Generalization in quantitative and qualitative research: Myths and strategies. International Journal of Nursing Studies, 47(11), 1451-1458. https://doi.org/https://doi.org/10.1016/j.ijnurstu.2010.06.004

VERSION 2 – REVIEW

**REVIEWER**	Lee, A<br>Monash Unversity , Physiotherapy
**REVIEW RETURNED**	25-Jun-2024

**GENERAL COMMENTS**	The authors have adequately addressed my comments and justified the included sample and commentary on data saturation aligning with the study aim.
